# Transcriptome profile and clinical characterization of ICOS expression in gliomas

**DOI:** 10.3389/fonc.2022.946967

**Published:** 2022-10-06

**Authors:** Jin Wang, Fei Shi, Aijun Shan

**Affiliations:** Department of Emergency, Shenzhen People’s Hospital (The Second Clinical Medical College, Jinan University; The First Affiliated Hospital, Southern University of Science and Technology), Shenzhen, China

**Keywords:** ICOS, immune response, prognosis, glioma, Treg

## Abstract

Inducible co-stimulator (ICOS), an immune costimulatory molecule, has been found to play an essential role across various malignancies. This study investigated the transcriptome profile and clinical characterization of ICOS in gliomas. Clinical information and transcriptome data of 301 glioma samples were downloaded from the Chinese Glioma Genome Atlas (CGGA) dataset for analysis (CGGA301 cohort). Furthermore, the results were validated in 697 samples with RNAseq data from the TCGA glioma dataset and 325 gliomas with RNAseq data from the CGGA325 dataset. Immunohistochemistry was performed to evaluate ICOS protein expression across different WHO grades in a tissue microarray (TMA). In addition, single-cell sequencing data from CGGA and GSE 163108 datasets were used to analyze the ICOS expression across different cell types. Statistical analyses and figure production were performed with R-language. We found that ICOS was significantly upregulated in higher-grade, IDH wild type, and mesenchymal subtype of gliomas. Functional enrichment analyses revealed that ICOS was mainly involved in glioma-related immune response. Moreover, ICOS showed a robust correlation with other immune checkpoints, including the PD1/PD-L1/PD-L2 pathway, CTLA4, ICOSL (ICOS ligand), and IDO1. Subsequent Tumor Immune Dysfunction and Exclusion (TIDE) analysis revealed that GBM patients with higher ICOS expression seemed to be more sensitive to ICB therapy. Furthermore, based on seven clusters of metagenes, GSVA identified that ICOS was tightly associated with HCK, LCK, MHC-I, MHC-II, STAT1, and interferon, especially with LCK, suggesting a strong correlation between ICOS and T-cell activity in gliomas. In cell lineage analysis, Higher-ICOS gliomas tended to recruit dendritic cells, monocytes, and macrophages into the tumor microenvironment. Single-cell sequencing analysis indicated that ICOS was highly expressed by regulatory T cells (Tregs), especially in mature Tregs. Finally, patients with higher ICOS had shortened survival. ICOS was an independent prognosticator for glioma patients. In conclusion, higher ICOS is correlated with more malignancy of gliomas and is significantly associated with Treg activity among glioma-related immune responses. Moreover, ICOS could contribute as an independent prognostic factor for gliomas. Our study highlights the role of ICOS in glioma and may facilitate therapeutic strategies targeting ICOS for glioma.

## Introduction

Gliomas, accounting for 70% of primary intracranial tumors in adult patients, are commonly characterized by a high mortality and disability rate (1). Despite substantial improvements in management, the unfavorable outcomes for gliomas remain unchanged, especially for the most aggressive type—GBM (2, 3); the median survival time remains less than 15 months. In the last decades, substantial immunotherapy advancements in other cancers have brought new hope for glioma treatment. Multiple studies have identified immune targets for glioma immunotherapy, mainly focusing on co-inhibitory checkpoints, including PD1/PD-L1 and CTLA4. However, most gliomas failed to respond to current immunotherapy, which facilitated us finding additional immune checkpoints for gliomas.

Inducible co-stimulator (ICOS, also termed CD278, H4, and AILIM), a member of the costimulatory molecule family consisting of ICOS, CD28, and CTLA4, is abundantly expressed on the cell surface of the activated and mature T cells rather than naïve T cells (4, 5). The costimulatory signal is induced after ICOS engagement with its unique ligand (ICOSL) to facilitate a series of immune-related biological processes, including development of germinal centers (6), activation of T-cell-dependent B cells, and switching of antibody class (7). More importantly, the ICOS/ICOSL pathway is particularly essential for T cells themselves, promoting their differentiation, proliferation, activation, and survival (5, 8). In addition, ICOS enhances the secretion of multiple immune cytokines, including TNF-α, IL-4, IL-5, IL-6, IL-10, and IL-21 (4, 5, 9, 10). The abnormality of ICOS expression leads to a range of pathophysiological dysfunctions, such as immunodeficiency, opportunistic infection, and malignant tumors.

ICOS has been widely reported as an important immune checkpoint among various cancers, including melanoma, gastrointestinal and liver cancer, gynecological cancer, breast cancer, renal clear cell carcinoma, and Merkel carcinoma. However, the roles of ICOS across different types of malignancies are inconsistent, mainly due to the dualistic effect of ICOS in the tumor microenvironment. On the one hand, ICOS exerts its anti-tumor effect through the enhancement of CD4+ and CD8+ effector T cells (11, 12); on the other hand, ICOS significantly activates and upregulates regulatory T cells (Tregs) (13), which are a subpopulation of T cells that mainly function as an immunosuppressor and consequently facilitate the immune escape in tumors (14). Therefore, many studies sought to determine the correlation between ICOS and outcomes of malignant tumors, which, as expected, yielded contradictory results. For patients with melanoma (15–17), gastric (18, 19) and liver cancer (20), gynecological (21, 22) and breast cancer (23, 24), and renal clear cell carcinoma (25), higher ICOS expression predicted much worse survival. In contrast, among patients who suffered from colorectal cancer (26, 27) and follicular lymphoma (28), the upregulation of ICOS expression yielded a better prognosis.

As an important immune costimulatory molecule, ICOS has attracted more and more attention in both hematologic and solid malignancies. However, no comprehensive report on gliomas has been reported. Only one study performed by Gousias et al. (29) investigated the fraction of ICOS+ Treg *via* immune-cell count analysis on 29 glioma patients. To elucidate the ICOS expression profile and clinical characterization in all gliomas, we collected microarray data of 301 glioma samples from the Chinese Glioma Genome Atlas (CGGA) dataset (CGGA301) and performed this integrative analysis of ICOS among whole-grade gliomas. Subsequently, the results were further validated in 697 gliomas with RNAseq from the TCGA dataset and 325 patients with RNAseq from the CGGA325 dataset. Our study would be the first comprehensive report to demonstrate the molecular and clinical characterization of ICOS expression among pan-gliomas. We believe that ICOS will become a potential hotspot for glioma immunotherapy.

## Materials and methods

### Sample collection

Microarray data and corresponding clinical information of 301 glioma patients (WHO grades II to IV) were obtained from the CGGA (30) dataset (CGGA301 dataset, http://www.cgga.org.cn/). The validation cohort utilized the TCGA (http://cancergenome.nih.gov/) (31) and CGGA325 glioma dataset, which included 697 patients (WHO grades II to IV) with RNAseq data (RSEM-normalized, level 3) and 325 samples (WHO grades II to IV) with RNAseq data (RSEM normalized), respectively. CGGA301 and CGGA325 are completely different datasets and contain samples that do not overlap. A total of 1,323 glioma patients were included in this study. In addition, the single-cell RNAseq data (sc-RNAseq) of glioma patients were obtained from CGGA (32), which consisted of 6,148 cells collected from 73 regions of 14 patients. Furthermore, the sc-RNAseq dataset of GSE163108 (33), consisting of 25,256 glioma-infiltrating T cells from 31 adult patients, was downloaded from the GEO website. Of them, there were 3,277 CD4+ T cells, 21,502 CD8+ T cells, 89 cycling T cells, and 388 Tregs. The study was approved by the Ethics Committee of Shenzhen People’s Hospital, and written consents were waived due to the use of de-identified patient data from public datasets.

### Data preprocessing

For RSEM-normalized RNA sequencing data from TCGA and CGGA325 datasets, log2 transformation was performed before analysis, while microarray data from the CGGA301 dataset, which had already been preprocessed by CGGA project, were analyzed directly. Lower-grade gliomas (LGGs) and GBMs were separately extracted for analysis. According to the WHO 2021 classification scheme for central nervous system tumors, grade IV gliomas with IDH-mutant can no longer be regarded as GBMs. Thus, GBM cohorts in this study were defined as those only with IDH wild type. During the survival analysis, patients with overall survival (OS) of less than 30 days and those without OS information were excluded from the Kaplan–Meier curve and Cox regression analysis. For sc-RNAseq data from CGGA and GSE163108, the data provider had already excluded the low-quality genes and low-quality cells. The percentage of mitochondria-expressed genes was less than 5% in CGGA, and 10% in GSE163108, respectively.

### Genomic alteration

For the TCGA cohort, data on somatic mutations and somatic copy number alterations (CNAs) were collected from the cBioPortal website (http://www.cbioportal.org). Differential somatic mutations and CNAs were compared between the high-ICOS group and low-ICOS groups according to the median ICOS expression level and were visualized with the oncoplot function provided in the ComplexHeatmap R-package.

### Functional enrichment analysis

ICOS co-expressed genes were identified according to the correlation coefficients of Pearson correlation (CGGA) or Spearman correlation (TCGA). The correlation coefficient |*r*| > 0.5 was set as the filter criteria to screen out significantly correlated genes of ICOS in each dataset. Gene Ontology (GO) analyses were performed on the DAVID (34) website (2021 update version, https://david.ncifcrf.gov/) and presented by pheatmap.

### Gene set enrichment analysis

Hallmark gene sets (h.all.v7.5.1.symbols.gmt) were obtained from the gene set enrichment analysis (GSEA) (35) website (http://software.broadinstitute.org/). The gene order for GSEA was pre-ranked according to the correlation coefficient value with ICOS. GSEA was performed and visualized with the clusterprofiler package (36). The number of permutations was 1000. Normalized enrichment score (NES) > 1 and false discovery rate (FDR) < 0.25 were considered as significantly enriched in the gene set.

### Gene set variation analysis

Seven immune-related gene clusters consisting of 104 genes, which represented distinct inflammatory activities, were termed metagenes (37) (**Supplementary Table 1**). Subsequent gene set variation analysis (GSVA) (38) was used for evaluation of the metagene expression level. After Spearman correlation analysis, intercorrelations among ICOS and the seven metagenes were presented with the Corrgram R-package.

### Response to immunotherapy

To evaluate the response to immune checkpoint blockades (ICBs, anti-PD1, or anti-CTLA4) of glioma patients, we performed a Tumor Immune Dysfunction and Exclusion (TIDE, http://tide.dfci.harvard.edu) analysis, which is an advanced bioinformatics approach to identify the patients with potential response to ICBs based on transcriptome expression profiles (39). Upon analyzing ICB response, transcriptome data were scaled and normalized by gene expression in each dataset. Patients with TIDE score below zero were defined as ICB responders.

### Cell-type enrichment analysis

The infiltration level of tumor-infiltrating immune cells (TIICs), immune score, stroma score, and microenvironment score were computed using the XCELL (40) R package (http://xCell.ucsf.edu/), which is a reliable and precise method to characterize cell composition based on transcriptome expression profiles. The relationship between ICOS and cell infiltration fraction in glioma was presented by pheatmap and corrplot packages.

### Single-cell RNA analysis

Sc-RNAseq analysis was performed with the Seurat package. After data normalization, a total of 2,000 highly variable genes were identified *via* the FindVariableGenes function. Subsequent principal component analysis (PCA) was performed with RunPCA, followed by FindNeighbors and FindClusters to cluster cells with the resolution of 0.1 in CGGA and 0.2 in GSE163108. Finally, results were presented by the UMAP method, and cell markers were utilized for cell annotation. The expression level of ICOS and cell markers were visualized with VlnPlot. Single-cell pseudotime trajectory analysis on Tregs was performed with the Monocle package. Treg cells were divided into seven states with five main branches.

### Immunohistochemistry of tissue microarray

A tissue microarray (TMA), including 125 glioma samples, was produced by Outdo Biotech (Product ID: HBraG125PG01, Shanghai, China). After antigen retrieval in the PT Link IHC preprocessing system (DAKO, Denmark), the TMA was incubated with an anti-ICOS antibody (Abcam, ab105227; 1:50 dilution) overnight at 4°C. Afterward, the Autostainer Link 48 platform (DAKO, Denmark) and EnVisionTM FLEX+ (K8002, DAKO, Denmark) were employed for secondary antibody binding and color development with diaminobenzidine (DAB), followed by nuclear counterstaining with hematoxylin. Finally, the TMA results were captured with Aperio XT Slide Scanner and visualized with Aperio ImageScope software. Two pathologists independently evaluated the IHC results of TMA to identify negative or positive ICOS expression on each tissue point.

### Statistical analysis

R language, together with a series of R-packages, including ggplot2, pheatmap, pROC, circlize, corrgram, clusterprofiler, survival, survminer, and forestmodel, was utilized for statistical analyses and graphical work. Gaussian distribution test was performed before data analysis. When comparing different clinical features and pathological factors between high- and low-ICOS groups, Student’s *t*-test, Wilcoxon test, and chi-square test were used where appropriate. Pearson and Spearman correlations were performed to assess the linear correlations between continuous variables. Kaplan–Meier survival analysis was performed to generate survival curves, followed by log-rank test to evaluate the statistical difference between groups. Cox regression analysis was performed with the coxph function provided in the survival package and visualized with the forestmodel package. All statistical tests were two-sided. A *p*-value less than 0.05 was considered statistically significant.

## Results

### ICOS is associated with clinical features and malignant phenotypes of glioma

**Figure 1:**
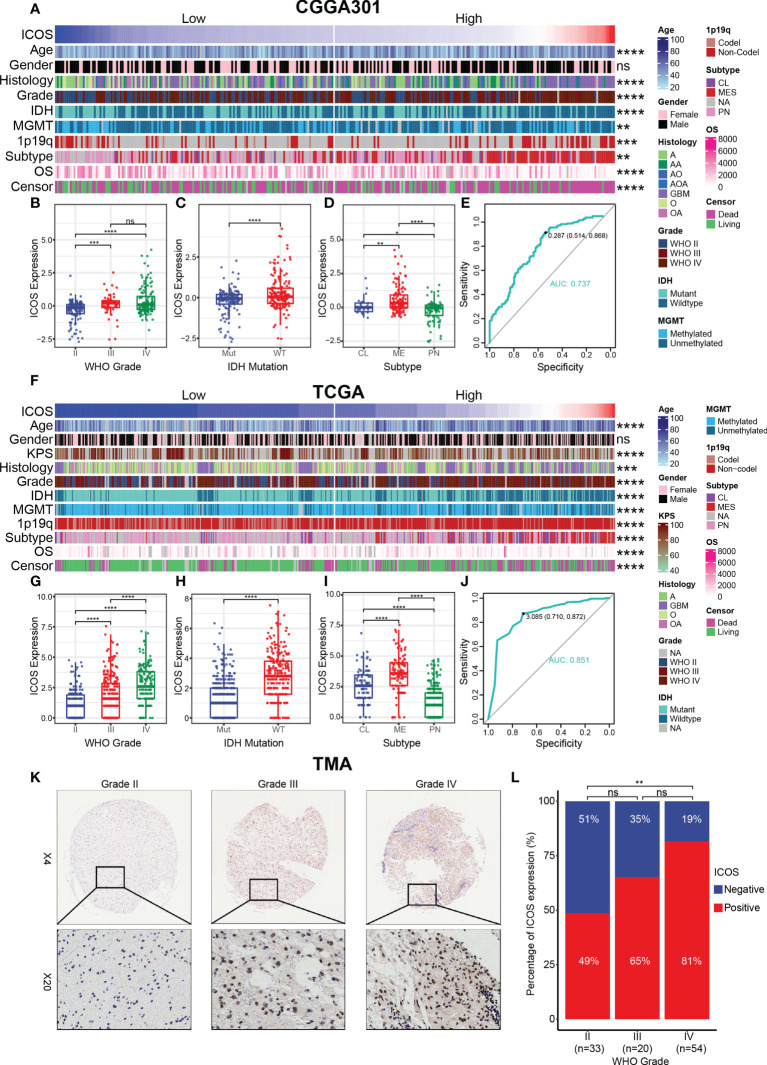
Relationship between ICOS expression and pathological characteristics. **(A)** The distribution of clinical and pathological characteristics arranged by the increasing ICOS expression in the CGGA301 dataset. **(B–D)** Distribution of ICOS expression in patients stratified by WHO grade, IDH mutation status, and TCGA molecular subtype in the CGGA301 dataset. **(E)** ROC curve to evaluate the sensitivity and specificity of ICOS expression to predict the mesenchymal subtype glioma in the CGGA301 dataset. **(F)** The distribution of clinical and pathological characteristics arranged by the increasing ICOS expression in the TCGA dataset. **(G–I)** Distribution of ICOS expression in patients stratified by WHO grade, IDH mutation status, and TCGA molecular subtype in the TCGA dataset. **(J)** ROC curve to evaluate the sensitivity and specificity of ICOS expression to predict the mesenchymal subtype glioma in the TCGA dataset. **(K)** Representative images of IHC staining for ICOS across different WHO grades of gliomas. **(L)** Comparison of IHC staining distribution (ICOS-negative or ICOS-positive) in different WHO grades of gliomas. * indicates *p* value < 0.05, ** indicates *p* value < 0.01, *** indicates *p* value < 0.001, **** indicates *p* value < 0.0001. ns, not significant.

According to the ICOS expression, the patients were arranged, and the landscape of corresponding clinical features and pathological characteristics was shown (**Figures 1A, F**, **Supplementary Figure 1A**). The results indicated that higher ICOS expression was significantly associated with elderly patients, low Karnofsky Performance Score (KPS), high WHO grade, IDH wild type, MGMT promoter unmethylation, 1p/19q non-codeletion, and shorter OS. Furthermore, ICOS expression levels among the WHO grades were compared. Similar results were obtained from three datasets and revealed that a higher grade was usually paralleled with an increased ICOS expression. Though no significant difference was detected between grades III and IV in CGGA301 and CGGA325 datasets, an increasing trend of ICOS could also be observed **(Figures 1B, G**, **Supplementary Figure 1B**). These findings suggested that ICOS upregulation was associated with more malignancy in gliomas. Moreover, IDH wild-type gliomas were found to be associated with a higher ICOS expression pattern compared to IDH-mutant counterparts in three datasets (**Figures 1C, H**, **Supplementary Figure 1C**), which further confirmed the correlation between ICOS and the aggressiveness in gliomas. Furthermore, ICOS expression was compared across different molecular subtypes. As shown in **Figures 1D, I**, ICOS was significantly upregulated in the mesenchymal than that in other subtypes, suggesting the potential discriminatory power of ICOS for mesenchymal-subtype gliomas. ROC curves showed that AUCs were 73.7% (**Figure 1E**) in CGGA301 and 85.1% (**Figure 1J**) in TCGA.

To confirm that ICOS expression was also upregulated at the protein level, we performed IHC staining for ICOS on a glioma TMA. Overall, the IHC staining intensity of ICOS was low-moderate in glioma tissues. Following exclusion of low-quality tissue points, there were 34 samples with negative IHC (34/107, 32%) and 73 samples with positive IHC (73/107, 68%), suggesting a significant heterogeneity of ICOS protein expression among different patients. The percentage of ICOS expression status was compared across different WHO grades (**Figures 1K, L**). The proportion of ICOS-positive status seemed to exhibit an increasing trend with the increasing level of WHO grade (II: 49%, III: 65%, and IV: 81%). No statistically significant differences were observed between grades II and III or between grades III and IV, which might be accounted for the small sample size in the grade III group. Generally, these results suggested that ICOS was closely correlated with the clinical characteristics and malignant features of glioma based on comprehensive transcriptomic and proteomic analyses.

### ICOS is relevant to distinct genomic alterations

**Figure 2:**
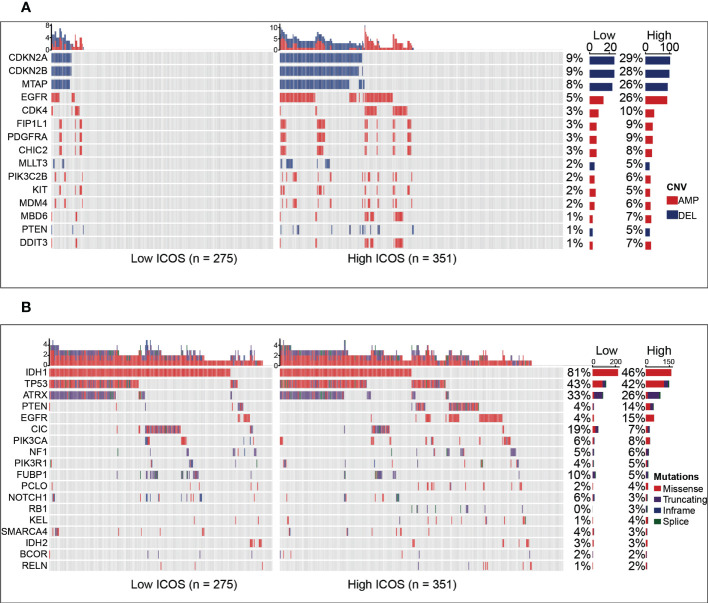
Comparison of genomic alterations between the high- and low-ICOS groups in the TCGA dataset. **(A)** Differential copy number variations (CNVs) between the high- and low-ICOS groups. **(B)** Differential somatic mutations between the high- and low-ICOS groups. AMP, amplification; DEL, deletion.

To estimate the correlations between the expression of ICOS and genomic characteristics in gliomas, somatic mutations and CNA analysis were performed using the TCGA dataset. An overall CNA profile was generated according to the comparison of high ICOS expression cluster vs. low ICOS expression cluster (**Figure 2A**). Generally, the high ICOS expression cohort showed high CNA frequency, with more deletion in tumor-suppressive genes, including CDKN2A, CDKN2B, MTAP, MLLT3, and PTEN, while with more amplification in oncogenic genes, including EGFR, CDK4, FIP1L1, PDGFRA, CHIC2, PIK3C2B, KIT, MDM4, MBD6, and DDIT3. Moreover, somatic mutation analysis based on ICOS expression revealed that more frequent mutations of PTEN, EGFR, RB1, and KEL were observed in the high-ICOS expression group, while mutations of IDH1, ATRX, CIC, FUBP1, and NOTCH1 were more frequently found in the low-ICOS expression group (**Figure 2B**). These findings indicate that gliomas with different ICOS expression levels show different genomic alterations.

### ICOS is involved in glioma-related immune response

**Figure 3:**
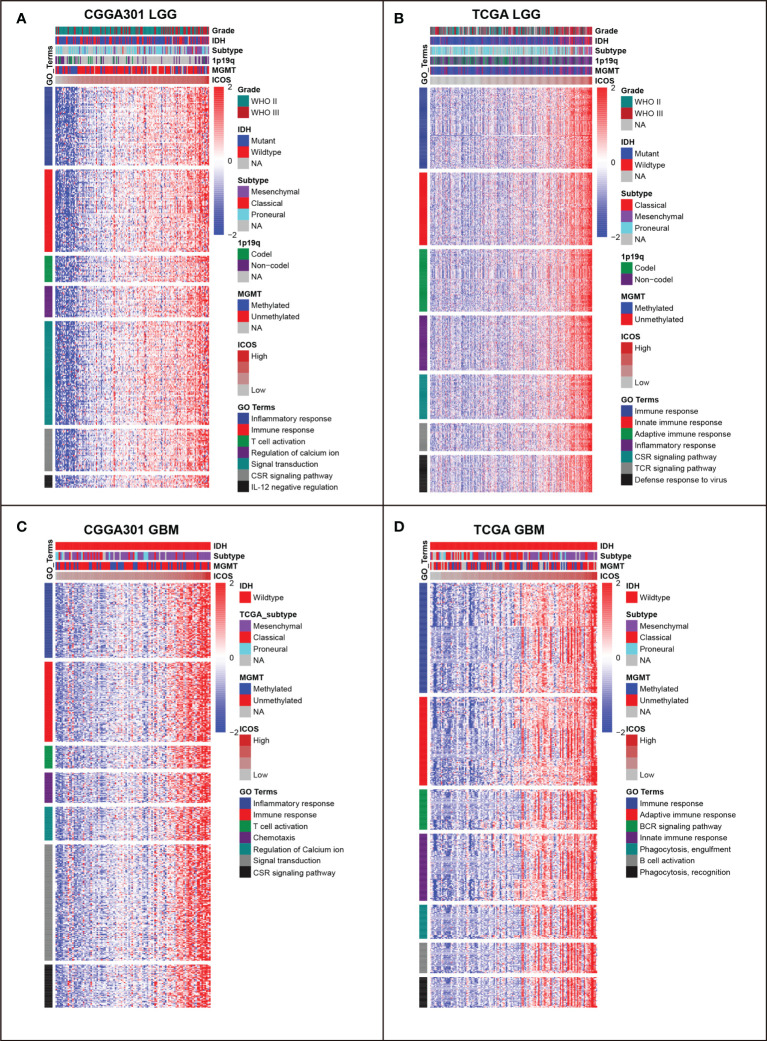
Gene ontology (GO) of genes associated with ICOS in CGGA301 and TCGA datasets. **(A)** GO in CGGA301 lower-grade glioma. **(B)** GO in TCGA lower-grade glioma. **(C)** GO in CGGA301 glioblastoma. **(D)** GO in TCGA glioblastoma.

To identify the biological functions related to ICOS, ICOS co-expressed genes were screened out for GO analysis. For the LGG cohort, there were 821 ICOS co-expressed genes in the CGGA301 dataset, 561 in the TCGA dataset, and 263 in the CGGA325 dataset. All co-expressed genes were positively correlated with ICOS and were mainly involved in a range of immune-related biological processes, including immune response, inflammatory response, innate immune response, adaptive immune response, and T-cell activation (**Figures 3A, B**, **Supplementary Figure 2A**). For the GBM cohort, we further evaluated ICOS-related biological processes in GBM. There were 1,463 ICOS co-expressed genes in the CGGA301 dataset, 451 in the TCGA dataset, and 290 in the CGGA325 dataset. Significantly correlated genes of three cohorts were further annotated, and we found that ICOS showed an even higher correlation with immune response and T-cell activation (**Figures 3C, D**, **Supplementary Figure 2B**). These results indicated that ICOS was mainly involved in glioma-related immune response.

**Figure 4:**
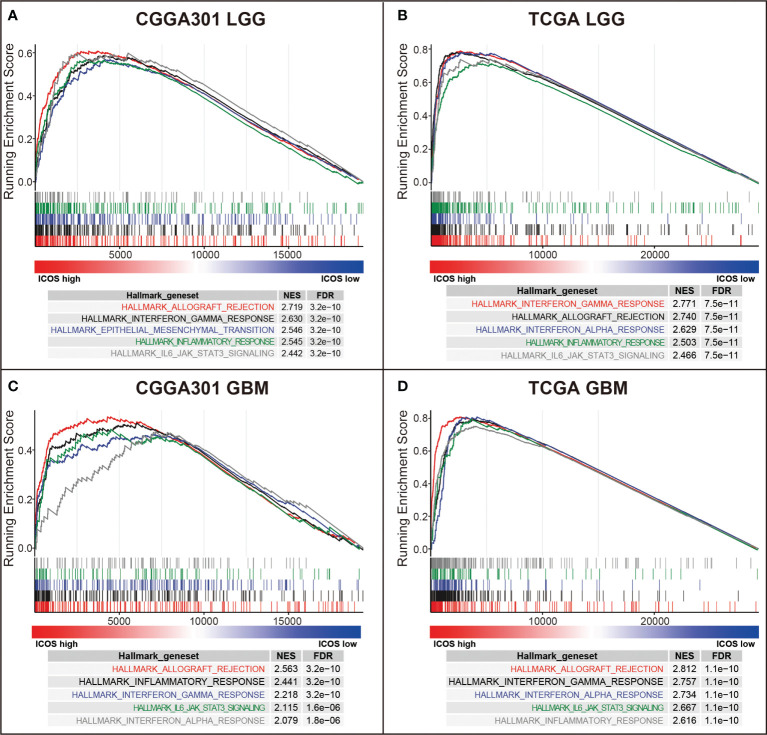
Gene set enrichment analysis (GSEA) of ICOS in CGGA301 and TCGA datasets. **(A)** GSEA in CGGA301 lower-grade glioma. **(B)** GSEA in TCGA lower-grade glioma. **(C)** GSEA in CGGA301 glioblastoma. **(D)** GSEA in TCGA glioblastoma.

GSEAs using the Hallmark gene sets were further performed to validate the ICOS-related biological process. In the LGG cohort, ICOS showed robust positive correlation with allograft rejection in CGGA301 (NES = 2.719, FDR < 0.001) (**Figure 4A**) followed by other hallmark gene sets, including interferon-gamma response (NES = 2.630, FDR < 0.001), epithelial–mesenchymal transition (NES = 2.546, FDR < 0.001), inflammatory response (NES = 2.545, FDR < 0.001), and IL6-JAK-STAT3 signaling (NES = 2.442, FDR < 0.001), which strongly pointed to the participation of ICOS in the glioma-related immune response. The results were further validated in TCGA and CGGA325 LGG cohorts (**Figure 4B**, **Supplementary Figure 3A**). In the CGGA301 GBM cohort, ICOS-associated genes were significantly enriched in allograft rejection (NES = 2.563, FDR < 0.001), inflammatory response (NES = 2.441, FDR < 0.001), interferon-gamma response (NES = 2.218, FDR < 0.001), IL6-JAK-STAT3 signaling (NES = 2.115, FDR < 0.001), and interferon-alpha response (NES = 2.079, FDR < 0.001) (**Figure 4C**). Moreover, we observed a similar pattern of GSEA results in TCGA and CGGA325 GBM cohorts (**Figure 4D**, **Supplementary Figure 3B**). These GSEA results further confirmed the profound association of ICOS with glioma-related immune response, consistent with what we observed in GO analysis.

### ICOS is associated with immune checkpoints and predicts response to immunotherapy

**Figure 5:**
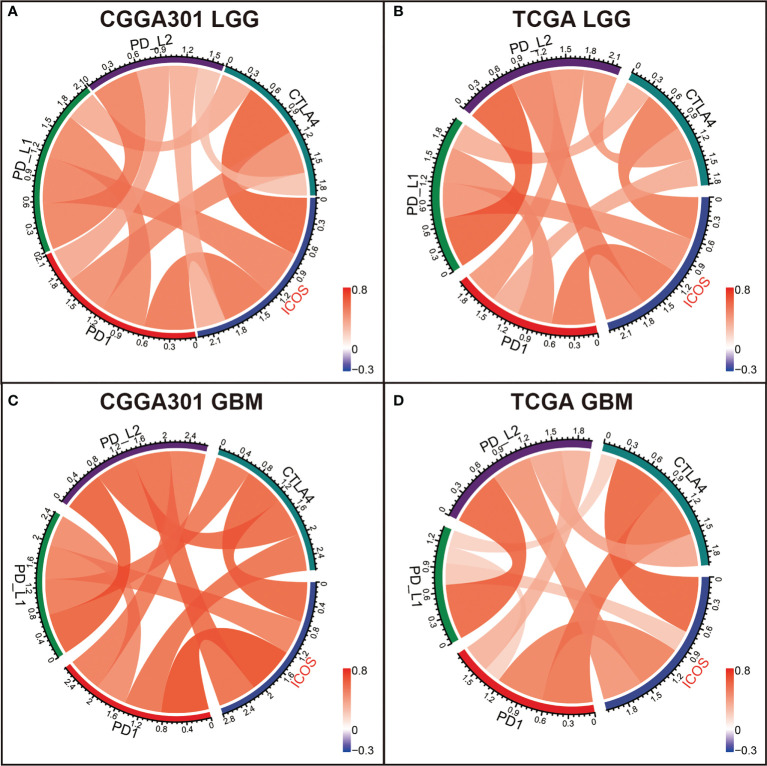
Correlation analysis between ICOS and canonical immune checkpoints in CGGA301 and TCGA datasets. **(A)** Correlation analysis in the CGGA301 dataset. **(B)** Correlation analysis in TCGA lower-grade glioma. **(C)** Correlation analysis in CGGA301 glioblastoma. **(D)** Correlation analysis in TCGA glioblastoma.

To further validate the interactions between ICOS and immune response, we performed correlation tests to explore the relationship between ICOS and a series of immune checkpoints, including PD-1, PD-L1, PD-L2, and CTLA-4. For the LGG cohort, Circos plots showed that ICOS level was positively correlated with all of these immune checkpoints among three datasets (**Figures 5A, B**, **Supplementary Figure 4A**), exhibiting synergistic interactions of these immune checkpoints in gliomas. For the GBM cohort, correlation tests were additionally performed to determine the relationship among these checkpoints in GBM. It was found that they exhibited more significant correlations with each other (**Figures 5C, D**, **Supplementary Figure 4B**). Multiple other checkpoints, such as TIM3, CD28, and IDO1, have been identified as therapeutic targets in preclinical experiments and clinical trials. We additionally examined the correlation between ICOS and these checkpoint members. ICOSLG, the ligand of ICOS, was also included in the analysis. Circos plots demonstrated that ICOS expression showed a robust correlation with ICOSLG and IDO1 in both LGG and GBM (**Supplementary Figures 4C, D** and **5A–D**). These results reminded us that there might be a tight synergistic interaction between the ICOS/ICOSLG axis and these canonical immune checkpoints.

**Figure 6:**
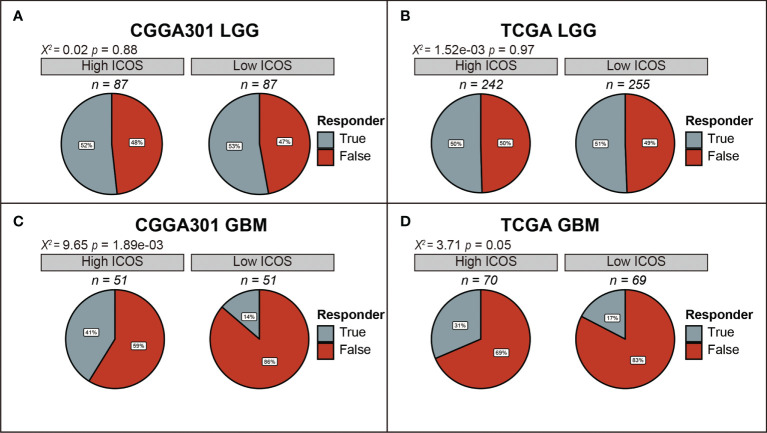
Comparison of response to immunotherapy between high-ICOS and low-ICOS groups in CGGA301 and TCGA datasets. **(A)** Response to immunotherapy in CGGA301 lower-grade glioma. **(B)** Response to immunotherapy in TCGA lower-grade glioma. **(C)** Response to immunotherapy in CGGA301 glioblastoma. **(D)** Response to immunotherapy in CGGA301 glioblastoma.

Furthermore, the TIDE algorithm was performed to estimate the ICB response in glioma. In the LGG cohort, there was no difference in ICB response between high- and low-ICOS groups (**Figures 6A, B**, **Supplementary Figure 6A**), while in the GBM cohort, patients with high ICOS levels seemed to be paralleled with a higher responding rate of ICB (CGGA301 *p* = 1.89e-03; TCGA *p* = 0.05; CGGA325 *p* = 4.23e-03) (**Figures 6C, D**, **Supplementary Figure 6B**), indicating that GBM with higher ICOS expression might be more sensitive to ICB therapy.

### ICOS-related inflammatory activities

**Figure 7:**
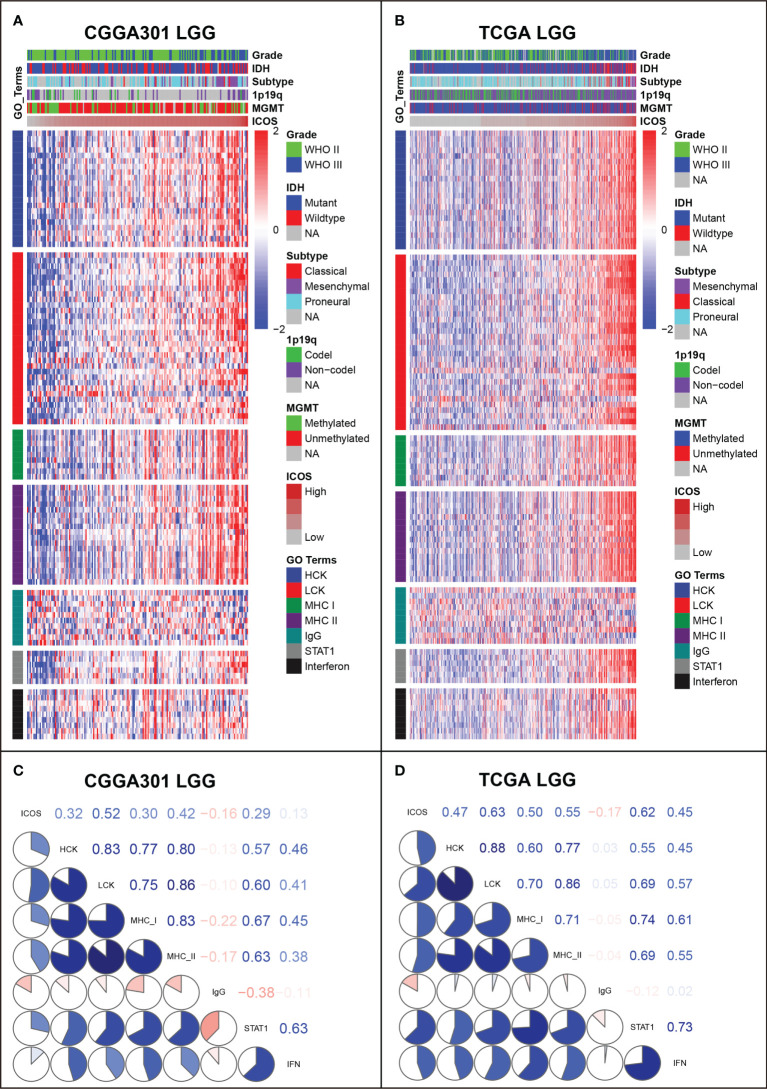
G**e**ne Set Variation Analysis (GSVA) of ICOS-related inflammatory activities of lower-grade glioma in CGGA301 and TCGA datasets. **(A)** Heatmap of representative genes from different inflammatory activities in CGGA301 lower-grade glioma. **(B)** Heatmap of representative genes from different inflammatory activities in TCGA lower-grade glioma. **(C)** Intercorrelation between ICOS and seven metagenes in CGGA301 lower-grade glioma. **(D)** Intercorrelation between ICOS and seven metagenes in TCGA lower-grade glioma.

**Figure 8:**
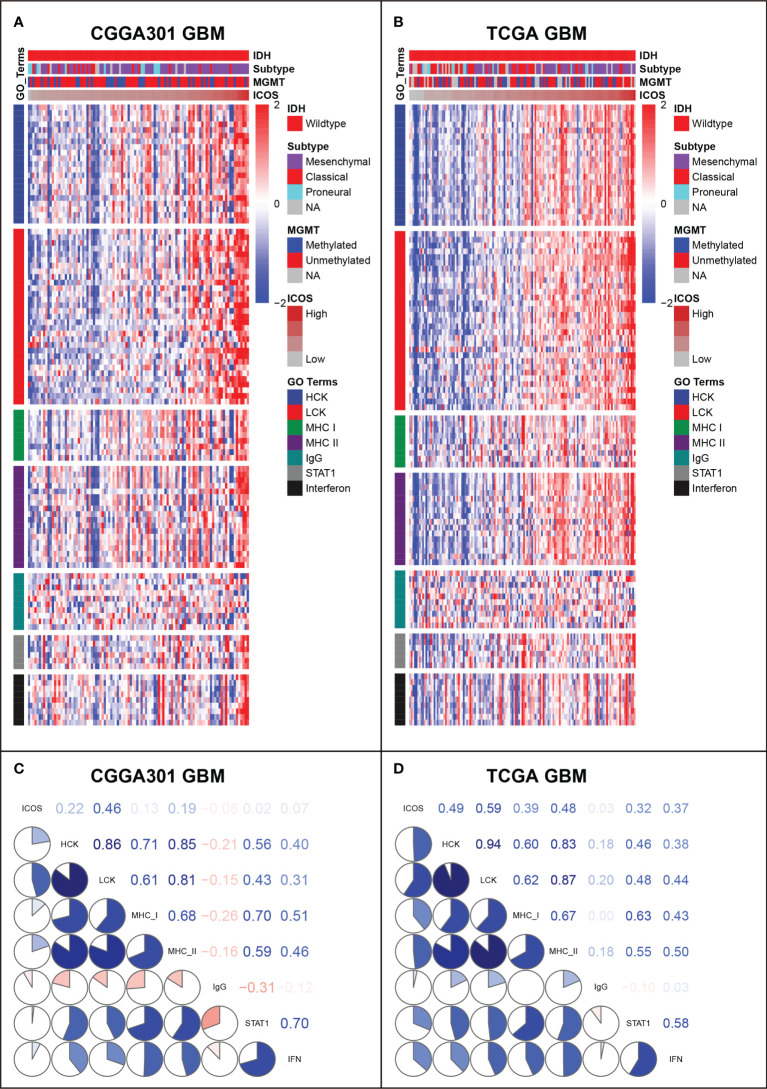
Gene Set Variation Analysis (GSVA) of ICOS-related inflammatory activities of glioblastoma in CGGA301 and TCGA datasets. **(A)** Heatmap of representative genes from different inflammatory activities in CGGA301 glioblastoma. **(B)** Heatmap of representative genes from different inflammatory activities in TCGA glioblastoma. **(C)** Intercorrelation between ICOS and seven metagenes in CGGA301 glioblastoma. **(D)** Intercorrelation between ICOS and seven metagenes in TCGA glioblastoma.

GSVA was performed to identify ICOS-related inflammatory activities. In the LGG cohort, ICOS expression revealed a significant correlation with most of the clusters, except for IgG, which specifically represented the immune activities of B cells (**Figures 7A, B**, **Supplementary Figure 7A**). Subsequently, correlation tests were performed between the expression levels of seven metagenes and ICOS. As shown in Corrgram plots (**Figures 7C, D**, **Supplementary Figure 7C**), ICOS was significantly positively correlated with HCK, LCK, MHC-I, MHC-II, STAT1, and Interferon, particularly with LCK, consistent with what we observed in clusters. We observed a similar pattern in GBM of all three datasets (**Figures 8A–D**, **Supplementary Figures 7B, D**). These findings enlightened us that ICOS might be profoundly associated with T-cell activities in gliomas.

### Relationship between ICOS and immune cell subpopulations in tumor microenvironment

**Figure 9:**
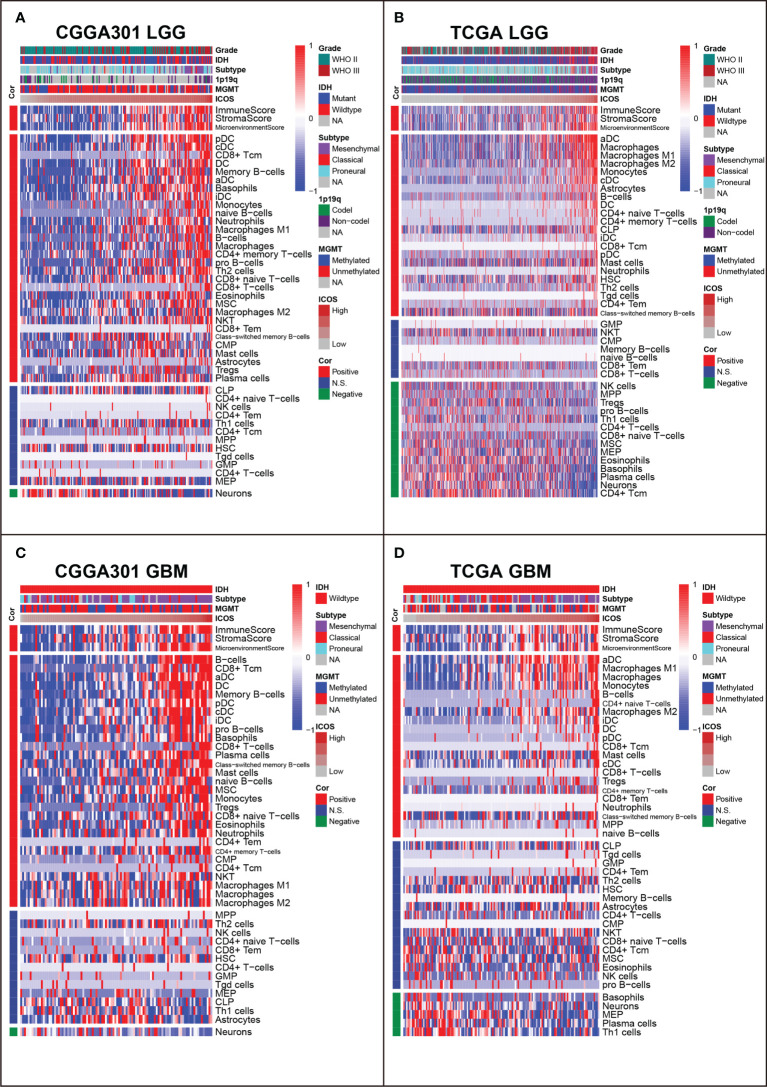
Relationship between ICOS and immune cell subpopulations in CGGA301 and TCGA datasets. **(A)** Correlation between ICOS and immune cell subpopulations in CGGA301 lower-grade glioma. **(B)** Correlation between ICOS and immune cell subpopulations in TCGA lower-grade glioma. **(C)** Correlation between ICOS and immune cell subpopulations in CGGA301 glioblastoma. **(D)** Correlation between ICOS and immune cell subpopulations in TCGA glioblastoma.

XCELL analysis (40) was performed to evaluate the immune score, stroma score, microenvironment score, and the cell subpopulations that ICOS might influence in glioma. In both LGG and GBM cohorts, we found that ICOS was consistently positively correlated with immune score, stroma score, and microenvironment score in three datasets. Through cell subpopulation enrichment analysis, ICOS showed a robust correlation with a series of antigen-presenting cells, including dendritic cells (DC), CD8+ T cells, monocytes, and macrophages. ICOS was negatively associated with normal brain neurons (**Figures 9A–D**, **Supplementary Figures 8A, B**). Notably, in LGG, the correlation between ICOS and Tregs was inconsistent across three datasets, while ICOS was significantly positively correlated with Tregs in three GBM cohorts. Overall, these results indicated that gliomas with higher ICOS tended to recruit multiple infiltrating immune cells into the tumor and were more associated with Tregs in more malignant gliomas.

To get a further understanding of ICOS-related immune cell composition, we selected canonical immune checkpoints, including PD1, PD-L1, PD-L2, and CTLA4, and compared them concurrently. Corrplots demonstrated that all these checkpoints were more associated with immune cell infiltration in GBM than those in LGG. All checkpoints were weakly correlated with CD4+ T cells except for PD-L2. ICOS was more associated with CD8+ T cells and Tregs than other checkpoints. In GBM, on the one hand, the correlation between ICOS and CD8+ T cells was enhanced, and on the other hand, the correlation with Tregs was synchronously increased, suggesting the dualistic role of ICOS in gliomas (**Supplementary Figures 9A–D**).

### ICOS is mainly expressed by regulatory T cells

**Figure 10:**
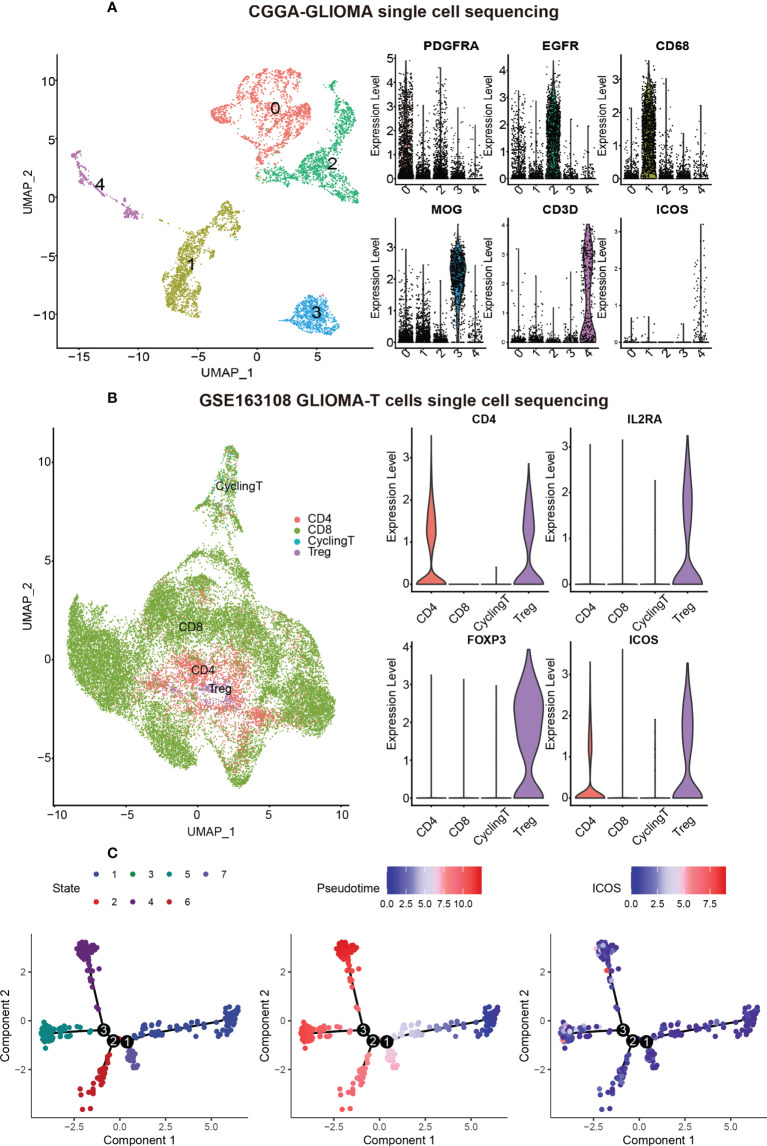
ICOS expression on different cell types based on single-cell RNAseq from CGGA and GSE163108 datasets. **(A)** Five cell clusters were identified in the CGGA sc-sequencing dataset. Clusters 0 and 2, highly expressing PDGFR and EGFR, could be annotated as glioma cells. Cluster 1, highly expressing CD68, was annotated as macrophages. Cluster 3, highly expressing MOG, could be identified as normal neurons. Cluster 4 highly expressing CD3D was annotated as T cells, and ICOS was mainly expressed in T cells rather than other cells. **(B)** Four types of glioma-infiltrated T cells were annotated by the data provider of GSE163108. We verified Treg’s cell markers (CD4, IL2RA [also termed CD25], and FOXP3), and ICOS was found to be particularly expressed in Tregs. **(C)** Single-cell trajectory analysis of Tregs reveals seven states. Cells are colored based on states (left), pseudotime (middle), and ICOS expression (right). State 1 could be identified as the early-stage Tregs, while states 4, 5, and 6 could be defined as the mature Tregs. ICOS is more upregulated in mature Tregs.

To identify the cell types that were highly expressing ICOS, CGGA sc-RNAseq data were analyzed (**Figure 10A**). Five cell clusters were annotated and visualized with the UMAP method. Based on the cell markers, cluster 0 overexpressing PDGFRA and cluster 2 overexpressing EGFR could be annotated as glioma cells. Cluster 1 expressing CD68 was annotated as monocyte–macrophage lineage, and cluster 4 expressing CD3D was concluded as T cells. Cluster 3, highly expressing MOG, represented a series of normal glial cells. As shown in **Figure 10A**, ICOS was found to be exclusively expressed by T cells (Cluster 4). To explore the T-cell subtypes correlated with ICOS upregulation, GSE163108 sc-RNAseq, focusing on T cells of gliomas, was further analyzed. Four types of glioma-infiltrating T cells, including CD8+ T, CD4+ T, Tregs, and cycling T cells, were captured and annotated by the data provider. We verified Tregs’ cell markers (CD4, IL2RA [CD25], and FOXP3). It turned out that ICOS was mainly expressed and activated on Tregs (**Figure 10B**), which further validated the immunosuppressive feature of ICOS among gliomas. Subsequent single-cell trajectory analysis of Tregs revealed seven states, among which state 1 was identified as the early-stage Tregs, while states 4, 5, and 6 could be defined as the mature Tregs. ICOS was more upregulated in mature Tregs (**Figure 10C**).

### ICOS predicts worse survival for gliomas

**Figure 11:**
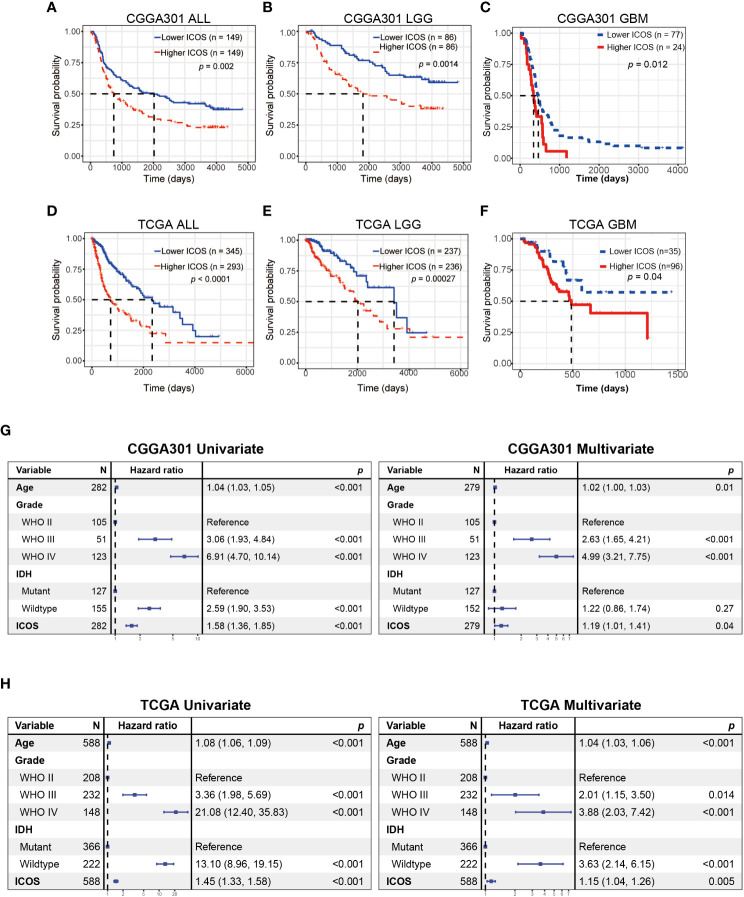
Survival analysis and Cox proportional hazards regression analysis according to ICOS expression. **(A–C)** Survival analysis in the CGGA301 dataset. **(D–F)** Survival analysis in the TCGA dataset. **(G)** Univariate and multivariate Cox regression model in the CGGA301 dataset. **(H)** Univariate and multivariate Cox regression model in the TCGA dataset.

Kaplan–Meier curves were delineated to explore the prognostic role of ICOS in gliomas. As results, patients with high ICOS levels exhibited significantly worse survival than patients with low ICOS (**Figures 11A, D**, **Supplementary Figure 10A**). Similar patterns were observed on the Kaplan–Meier curves of LGG (**Figures 11B, E**) and GBM patients (**Figures 11C, F**). Cox regression analysis was performed to investigate the independent prognostic role of ICOS, together with age, WHO grade, and IDH mutation, and the results revealed that higher ICOS was associated with an unfavorable prognosis independently (CGGA301: HR = 1.191, *p* = 0.044; TCGA: HR = 1.151, *p* = 0.005; CGGA325: HR = 1.57, *p* = 0.002) (**Figures 11G, H**, **Supplementary Figure 10D**). These findings suggested that ICOS could serve as an independent unfavorable prognostic factor for glioma patients.

## Discussion

Despite numerous efforts to improve prognosis, glioma remains the most common and lethal malignancy in the central nervous system. There is a pressing need to explore novel therapeutic strategies for gliomas. Immunotherapy brings new hope for the treatment of malignancies (41, 42). PD-1 and CTLA4 are two well-known immune checkpoints through which cancers prevent the immune system from recognizing and attacking tumor cells. Anti-PD-1 and anti-CTLA4 can re-activate the anti-tumor response, leading to tumor regression (43, 44). Unfortunately, anti-PD-1 and anti-CTLA4 monotherapy, although an important step in the right direction, was not as effective as it was hoped to be for gliomas (45). Combining existing immune checkpoint blockades and novel targets promises to achieve more success in immunotherapy for glioma patients. As an essential immune costimulatory member, ICOS aroused researchers’ interest because of its dualistic effect during oncogenesis across different tumors.

Our study provides the most comprehensive investigation of the expression profile of ICOS in whole WHO grade gliomas, together with its related molecular signature, clinical significance, and prognostic value. Through analysis of transcriptome expression status of ICOS among 1,323 glioma patients, we found that higher ICOS expression correlated with higher malignancy of gliomas, based on the fact that ICOS upregulation was remarkably paralleled with much more aggressive characteristics of gliomas, including glioblastoma, IDH wild type, and mesenchymal subtype, and could serve as an independent prognosticator for worse survival. These results indicated that ICOS precisely reflected the malignancy of gliomas and could play a pro-oncogenic role in the development and progression of glioma, consistent with the results reported by Iwata et al. (46). Through investigation of expression status and molecule function of the ligand of ICOS (ICOSLG) in glioblastomas, they concluded that ICOSL, *via* conjugation with ICOS, was associated with more malignancy of glioblastoma. In addition, our Circos plots also demonstrated the robust interaction between ICOS and ICOSLG in both LGG and GBM, further confirming the pro-oncogenic role of the ICOS/ICOSLG pathway in gliomas.

The involvement of ICOS in gliomagenesis was further explored through the perspective of genomic alterations. High ICOS expression level was found to be significantly correlated with high amplification in multiple oncogenic drivers, such as EGFR, PDGFRA, CDK4, PIK3C2B, and MDM4 (47), and with more frequent deletions in tumor-suppressing genes like PTEN, CDKN2A, and CDKN2B (48). Given the profound associations with distinct genomic alterations, ICOS is supposed to play a vital role in gliomagenesis, glioma progression, microenvironment remodeling, and drug resistance (49), further confirming its robust correlation with higher malignancy of gliomas.

Although the exact mechanisms of ICOS in the regulation of tumorigenesis and development of glioma are not well understood, some T-cell subpopulations might play essential roles in this process. Reportedly, the immunosuppressive effect of ICOS may be attributed to the presence of ICOS^+^ Tregs, accounting for 5%–10% of all peripheral CD4^+^ T cells. Tregs are a subpopulation of T cells that are involved in aspects of immunosuppression. Upregulation or activation of Tregs is associated with higher ICOS expression in tumor-infiltrating T cells (29, 46). In this study, through functional analysis of GO and GSEA, we found a consistent result that a higher ICOS level was associated with a more-activated immune response, especially with T-cell activity. It was reported that the number of Tregs increased in the glioma microenvironment, and downregulation of Tregs significantly enhanced the effect of immunotherapy (50). ICOS pathway not only promotes Tregs generation *via* mediating Th17-to-Treg transdifferentiation but also maintains the immunosuppressive effect of Tregs (14). Our sc-RNAseq analysis revealed that ICOS was significantly upregulated in Tregs rather than CD4+ and CD8+ T cells, confirming the immunosuppressive role of ICOS *via* upregulation and activation of Tregs, especially in mature Tregs. Through the immune cell subpopulation analysis, for LGG cohorts, ICOS showed a weak correlation with Tregs, while for GBM cohorts, ICOS exhibited a significant association with Tregs, suggesting that ICOS was more associated with Treg activation in GBM, in contrast to a relatively weak effect in LGG.

Our results demonstrated that ICOS showed a robust correlation with the PD1/PD-L1/PD-L2 pathway, CTLA-4, and indoleamine 2,3-dioxygenase 1 (IDO1), suggesting synergistic interactions among these immune checkpoints. Further TIDE analysis, aiming to predict response to ICB, revealed that patients with higher ICOS seemed to be more sensitive to ICB therapy in GBM cohorts, suggesting that ICOS can be employed to predict the response of GBM patients before anti-PD-1/anti-CTLA4 immunotherapy. Furthermore, therapies targeting the ICOS/ICOSL pathway in combination with existing immune checkpoint blockades might produce synergistic effects in efficacy, which have been evaluated in preclinical and clinical experiments. To date, four monoclonal antibodies (mAbs) targeting ICOS are evaluated in clinical trials, among which three are agonistic (GSK3359609, JTX-2011, and KY1044), and one is antagonistic (MEDI-570). The clinical trials mainly focused on anti-ICOS in combination with anti-PD1/PD-L1 or anti-CTLA4 (NCT02904226, NCT02520791, NCT03829501 NCT02723955, and NCT03251924) (51). Zamarin et al. (52) demonstrated that ICOS agonism in tumors enhanced the effectiveness of CTLA-4 blockade in the anti-tumor immune response. Despite activation in Tregs during the treatment, other anti-tumor T cells (CD8^+^ and CD4^+^) were more activated in the tumor microenvironment. Fan et al. (53) also reported a synergistic anti-tumor response of CTLA-4 blockade combined with ICOS stimulation in a mouse cancer model. However, anti-ICOS antagonist mAb also exhibited an anti-tumor effect in some malignancies, including follicular B-cell lymphoma (28) and prostate cancer (54), which may account for the decrease in Treg function and proliferation. Thus, considering the remarkable immunosuppressive effect of ICOS, an anti-ICOS antagonist might be more appropriate in glioma; however, this requires a further in-depth understanding of the mechanism of ICOS. Meanwhile, IDO1, a metabolic modulator reported to promote tumors developing immunotherapy resistance (55), has also been identified as an immune target, and the evaluation of anti-IDO1 is undergoing (56). ICOS showed a strong correlation with these molecules, providing more evidence for immunotherapy combination for gliomas.

In addition, the correlation between ICOS expression and seven inflammatory metagenes was further determined. These metagenes represented distinct immune perspectives. The weak correlation between ICOS and IgG was associated with a low abundance of B-lineage cells in the CNS. The other six ICOS-correlated metagenes mainly reflected the involvement of ICOS in the activities of T cells (LCK), antigen-presenting cells (HCK, MHC-I, and MHC-II), and interferon-response signaling pathway (STAT1 and IFN), which was in line with the results above. Moreover, we investigated the association between ICOS and immune score, stroma score, microenvironment score, and immune cell subpopulations. ICOS expression showed a positive correlation with the immune score, stromal score, and microenvironment score, and Higher-ICOS gliomas tended to recruit multiple infiltrating immune cell types. Given the robust correlation between ICOS and a broad range of immune activities revealed, ICOS is considered to influence the glioma-related immune response profoundly.

The studies performed by Gousias et al. (29), focusing on ICOS+ Tregs, and Iwata et al. (46), characterizing ICOSLG expression in GBM, have done an impressive job and elucidated the vital role of ICOS pathway in glioma and Tregs. Our study, mainly focusing on transcriptome characterization and clinical significance, extended the research in whole grades of glioma, further highlighted the essential role of ICOS among pan-gliomas, and profoundly expanded the spectrum of research on ICOS. In conclusion, our study reveals the potential of therapies targeting ICOS for glioma immunotherapy. Nevertheless, a limitation of the current study was that no experimental validation was performed. Further *in vitro* and *in vivo* studies are needed to validate the biological behaviors of ICOS in glioma. Moreover, future studies are warranted to evaluate the efficacy and safety of anti-ICOS in combination with other immune checkpoint inhibitors.

## Data availability statement

The original contributions presented in the study are included in the article/**Supplementary Material**. Further inquiries can be directed to the corresponding authors.

## Ethics statement

The studies involving human participants were reviewed and approved by Ethics Committee of Shenzhen People’s Hospital. Written informed consent for participation was not required for this study in accordance with the national legislation and the institutional requirements.

## Author contributions

AS and FS made contributions to the study conception and design. JW and FS participated in data acquisition and data analysis, drafted the manuscript, and revised it critically. All authors have read and approved the final manuscript.

## Funding

This work was supported by Shenzhen People’s Hospital (SYJCYJ202001).

## Acknowledgments

We sincerely thank the Chinese Glioma Genome Atlas (CGGA) team and Cancer Genome Atlas (TCGA) team for their generosity in sharing big data. They have made a substantial dedication to the scientific world and human health.

## Conflict of interest

The authors declare that the research was conducted in the absence of any commercial or financial relationships that could be construed as a potential conflict of interest.

## Publisher’s note

All claims expressed in this article are solely those of the authors and do not necessarily represent those of their affiliated organizations, or those of the publisher, the editors and the reviewers. Any product that may be evaluated in this article, or claim that may be made by its manufacturer, is not guaranteed or endorsed by the publisher.

## Supplementary material

Supplementary Figure 1 Relationship between ICOS expression and the pathological characteristics in CGGA325 dataset. **(A)** The distribution of clinical and pathological characteristics arranged by the increasing ICOS expression in CGGA325 dataset. **(B, C)** Distribution of ICOS expression in patients stratified by WHO grade and IDH mutation status in CGGA325 dataset.

Supplementary Figure 2 Gene ontology (GO) of genes associated with ICOS in CGGA325 dataset. **(A)** GO in CGGA325 lower-grade glioma. **(B)** GO in CGGA325 glioblastoma.

Supplementary Figure 3 Gene set enrichment analysis (GSEA) of ICOS in CGGA325 dataset. (A) GSEA in CGGA325 lower-grade glioma. (B) GSEA in CGGA325 glioblastoma.

Supplementary Figure 4 Correlation analysis between ICOS and other immune checkpoint members in CGGA325 dataset. **(A)** Correlation between ICOS and canonical immune checkpoints in lower-grade glioma. **(B)** Correlation between ICOS and canonical immune checkpoints in glioblastoma. **(C)** Correlation between ICOS and additional immune checkpoints in lower-grade glioma. **(D)** Correlation between ICOS and additional immune checkpoints in glioblastoma.

Supplementary Figure 5 Correlation analysis between ICOS and additional immune checkpoints in CGGA301 and TCGA datasets. **(A)** Correlation analysis in CGGA301 dataset. **(B)** Correlation analysis in TCGA lower-grade glioma. **(C)** Correlation analysis in CGGA301 glioblastoma. **(D)** Correlation analysis in TCGA glioblastoma.

Supplementary Figure 6 Comparison of response to immunotherapy between high ICOS and low ICOS groups in CGGA325 dataset. **(A)** Response to immunotherapy in lower-grade glioma. **(B)** Response to immunotherapy in glioblastoma.

Supplementary Figure 7 G**e**ne Sets Variation Analysis (GSVA) of ICOS-related inflammatory activities in CGGA325 dataset. **(A)** Heatmap of representative genes from different inflammatory activities in lower-grade glioma. **(B)** Heatmap of representative genes from different inflammatory activities in glioblastoma. **(C)** Intercorrelation between ICOS and seven metagenes in lower-grade glioma. **(D)** Intercorrelation between ICOS and seven metagenes in glioblastoma.

Supplementary Figure 8 Relationship between ICOS and immune cell subpopulations in CGGA325 dataset. **(A)** Correlation between ICOS and immune cell subpopulations in lower-grade glioma. **(B)** Correlation between ICOS and immune cell subpopulations in glioblastoma.

Supplementary Figure 9 Comparison of cell fraction correlation between ICOS and other checkpoints in CGGA301 and TCGA datasets.

Supplementary Figure 10 Survival analysis and Cox proportional hazards regression analysis according to ICOS expression in CGGA325 dataset. **(A–C)** Survival analysis in CGGA325 dataset. **(D)** Univariate and multivariate Cox regression model in CGGA325 dataset.
