# Supplementary figures and images for: Transcriptome profile and clinical characterization of ICOS expression in gliomas
Source: Front Oncol. 2022 Oct 6;12:946967. doi: 10.3389/fonc.2022.946967 (PMC9582985; doi:10.3389/fonc.2022.946967)

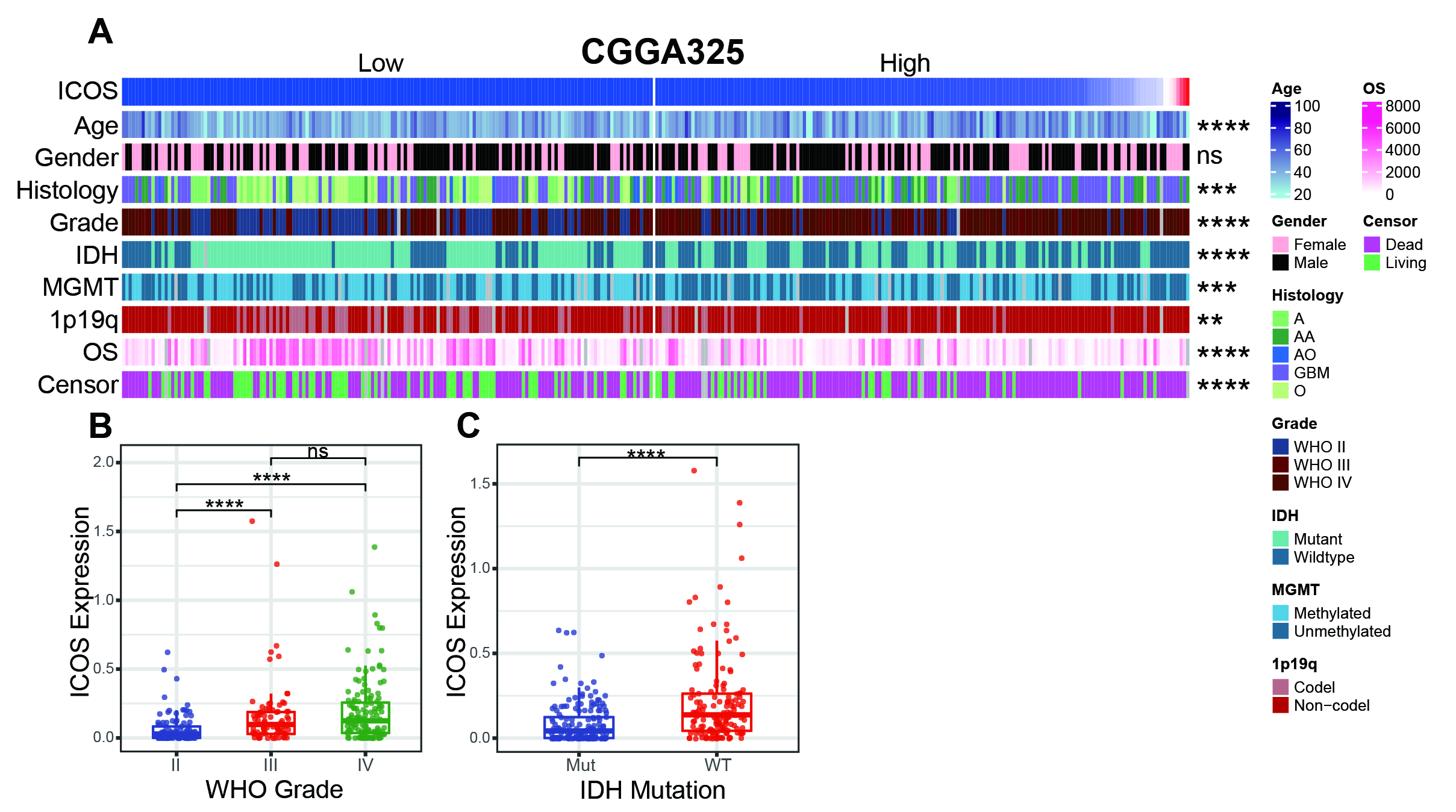

Supplement: Supplementary Figure 1 — Relationship between ICOS expression and the pathological characteristics in CGGA325 dataset. (A) The distribution of clinical and pathological characteristics arranged by the increasing ICOS expression in CGGA325 dataset. (B, C) Distribution of ICOS expression in patients stratified by WHO grade and IDH mutation status in CGGA325 dataset. [file Image_1.tif]

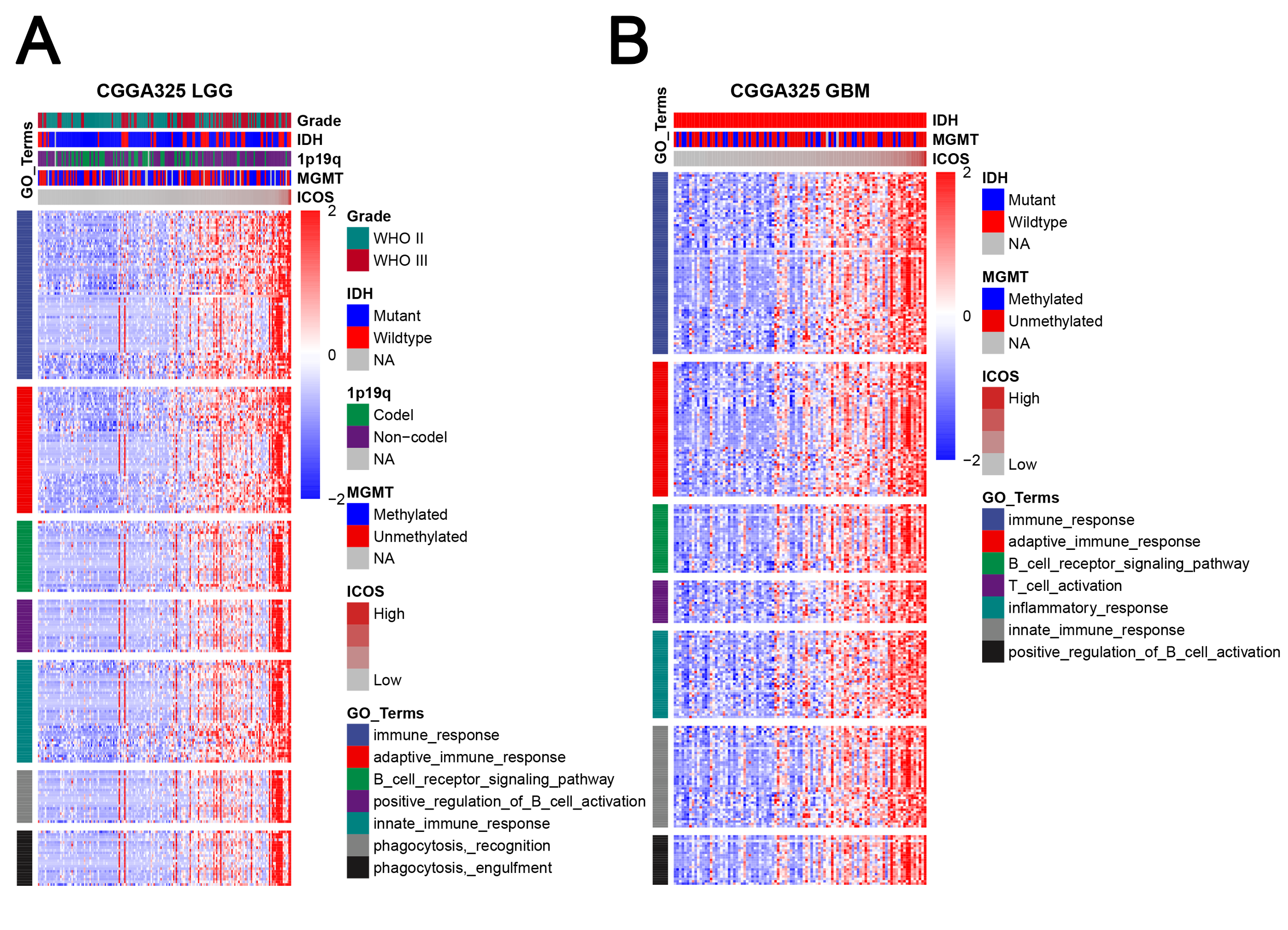

Supplement: Supplementary Figure 2 — Gene ontology (GO) of genes associated with ICOS in CGGA325 dataset. (A) GO in CGGA325 lower-grade glioma. (B) GO in CGGA325 glioblastoma. [file Image_2.tif]

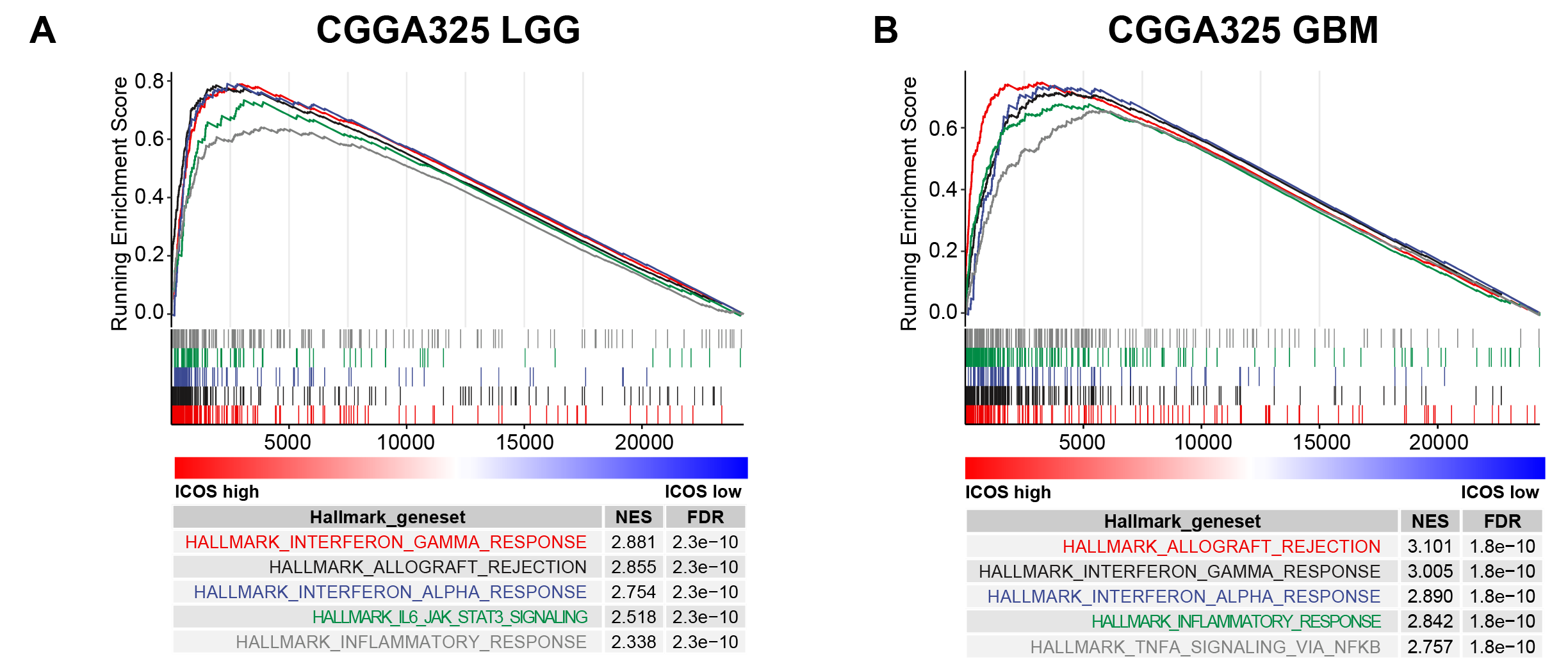

Supplement: Supplementary Figure 3 — Gene set enrichment analysis (GSEA) of ICOS in CGGA325 dataset. (A) GSEA in CGGA325 lower-grade glioma. (B) GSEA in CGGA325 glioblastoma. [file Image_3.tif]

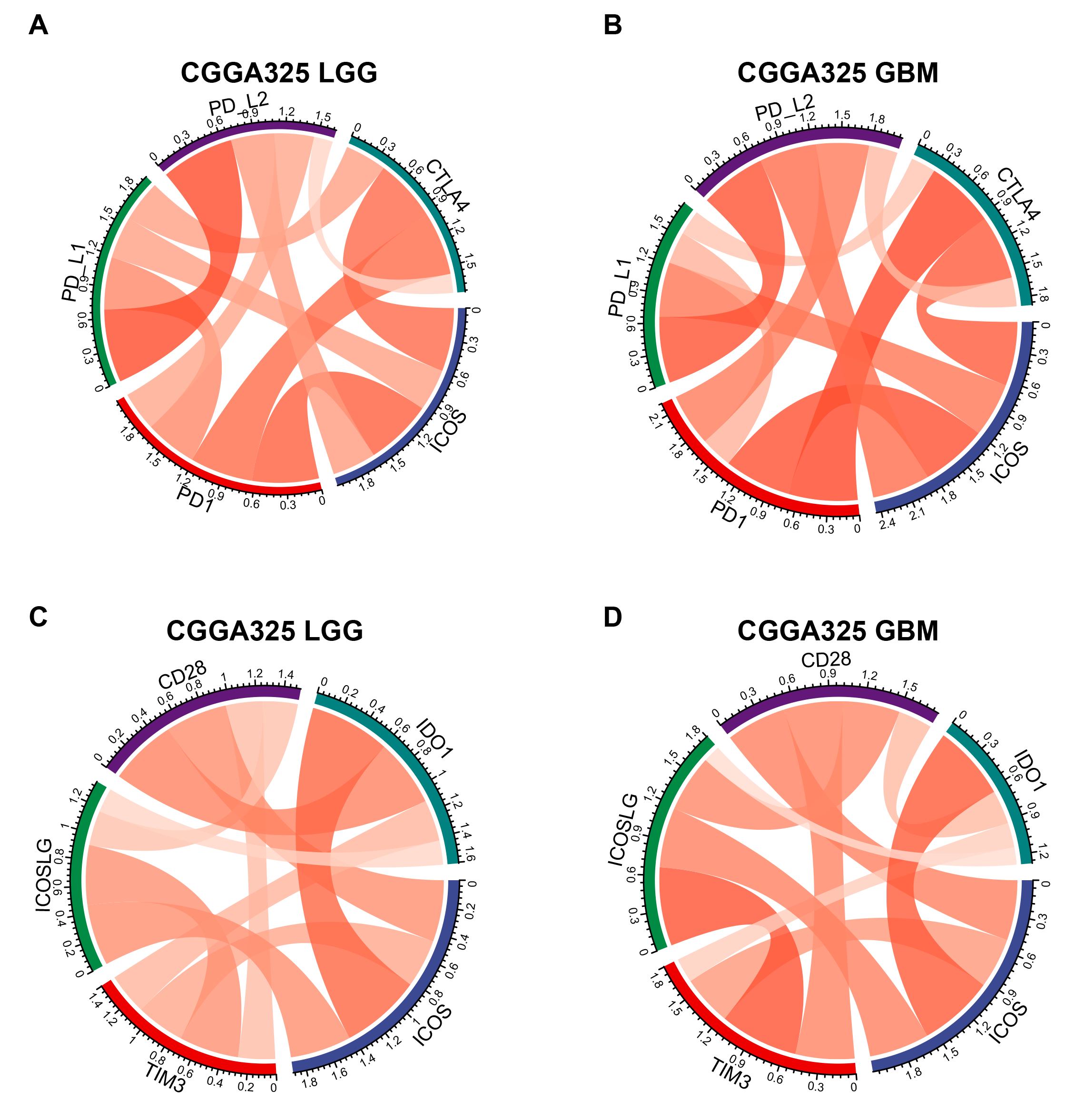

Supplement: Supplementary Figure 4 — Correlation analysis between ICOS and other immune checkpoint members in CGGA325 dataset. (A) Correlation between ICOS and canonical immune checkpoints in lower-grade glioma. (B) Correlation between ICOS and canonical immune checkpoints in glioblastoma. (C) Correlation between ICOS and additional immune checkpoints in lower-grade glioma. (D) Correlation between ICOS and additional immune checkpoints in glioblastoma. [file Image_4.tif]

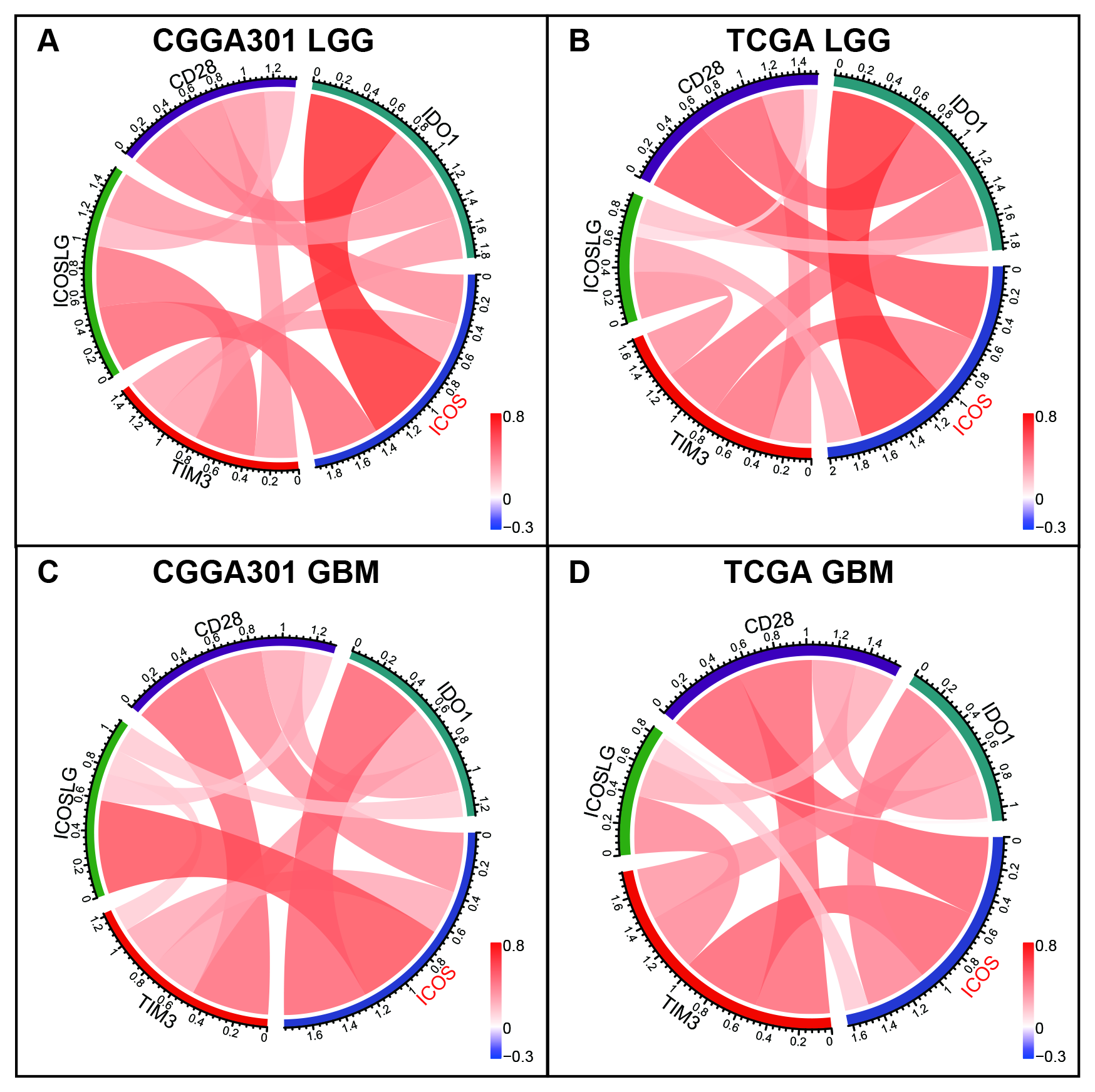

Supplement: Supplementary Figure 5 — Correlation analysis between ICOS and additional immune checkpoints in CGGA301 and TCGA datasets. (A) Correlation analysis in CGGA301 dataset. (B) Correlation analysis in TCGA lower-grade glioma. (C) Correlation analysis in CGGA301 glioblastoma. (D) Correlation analysis in TCGA glioblastoma. [file Image_5.tif]

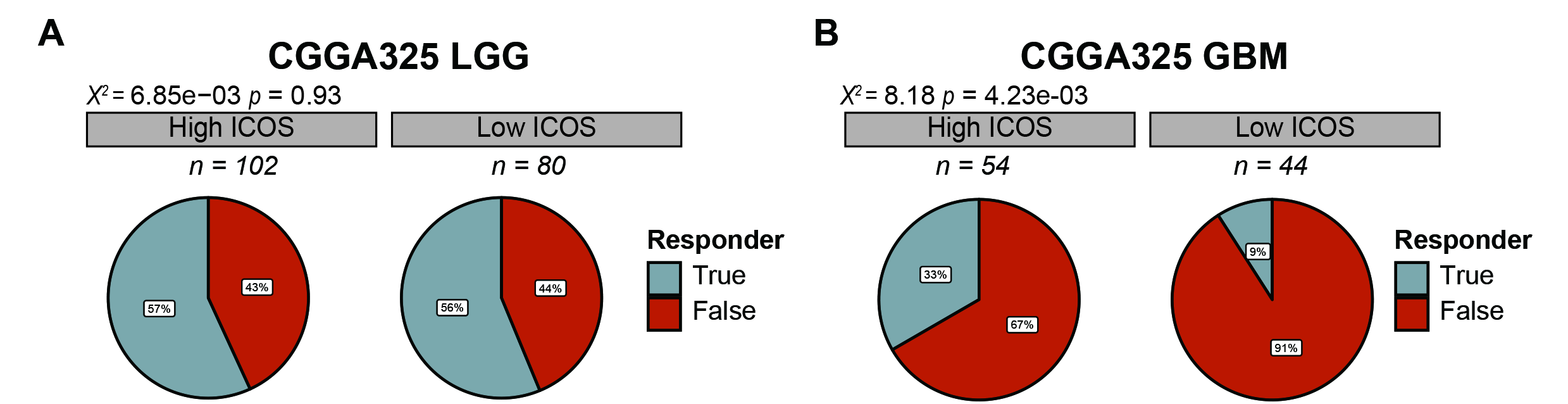

Supplement: Supplementary Figure 6 — Comparison of response to immunotherapy between high ICOS and low ICOS groups in CGGA325 dataset. (A) Response to immunotherapy in lower-grade glioma. (B) Response to immunotherapy in glioblastoma. [file Image_6.tif]

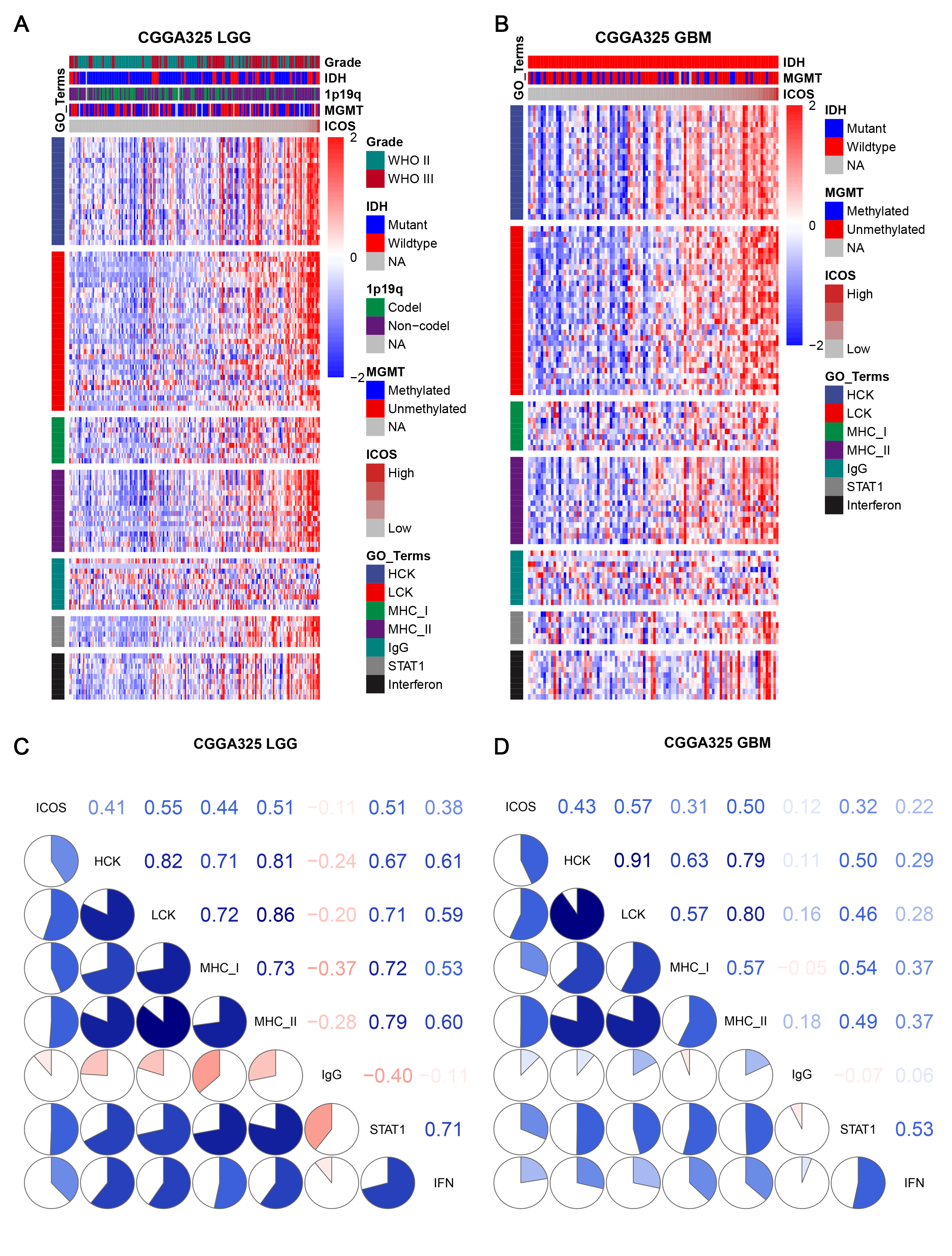

Supplement: Supplementary Figure 7 — Gene Sets Variation Analysis (GSVA) of ICOS-related inflammatory activities in CGGA325 dataset. (A) Heatmap of representative genes from different inflammatory activities in lower-grade glioma. (B) Heatmap of representative genes from different inflammatory activities in glioblastoma. (C) Intercorrelation between ICOS and seven metagenes in lower-grade glioma. (D) Intercorrelation between ICOS and seven metagenes in glioblastoma. [file Image_7.tif]

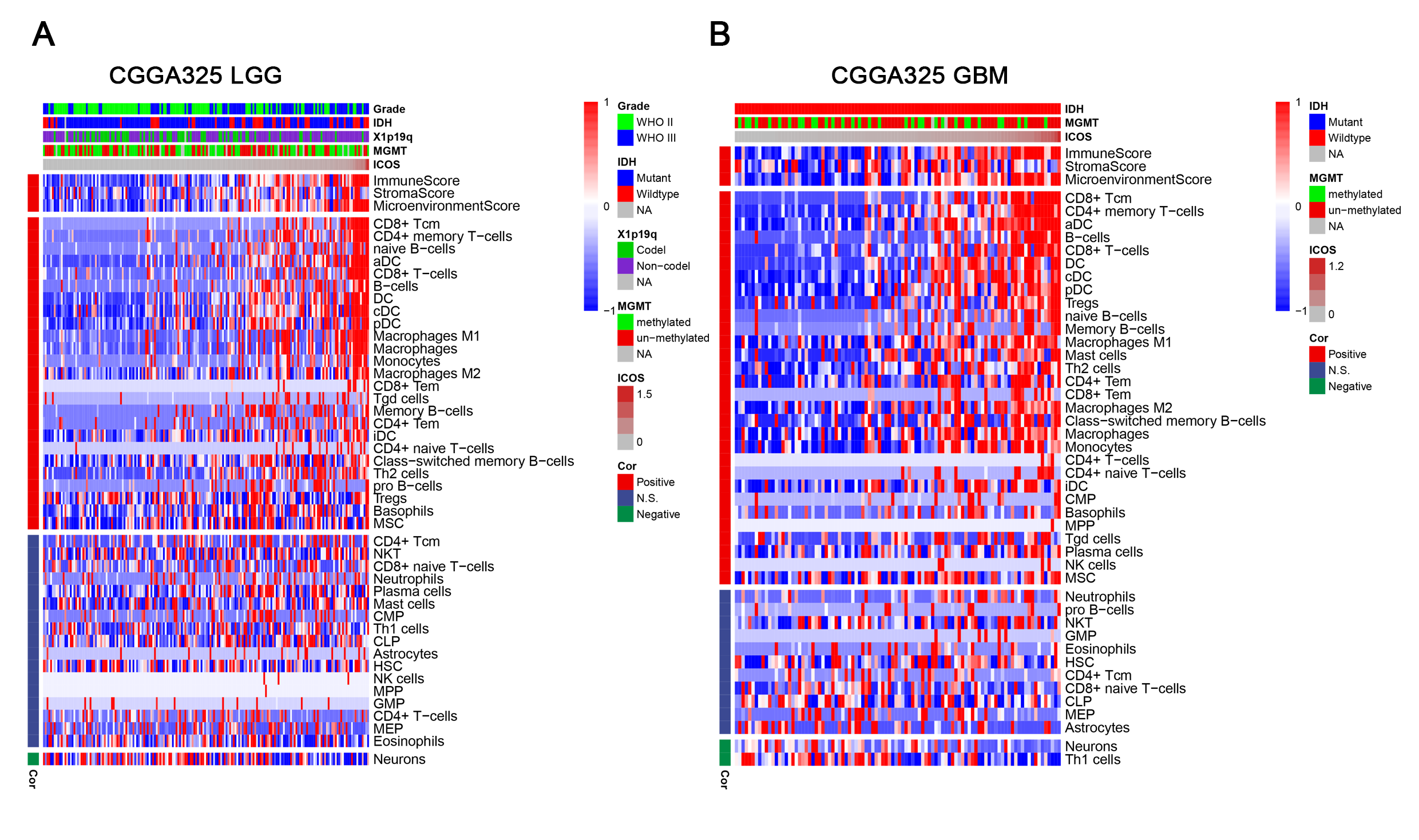

Supplement: Supplementary Figure 8 — Relationship between ICOS and immune cell subpopulations in CGGA325 dataset. (A) Correlation between ICOS and immune cell subpopulations in lower-grade glioma. (B) Correlation between ICOS and immune cell subpopulations in glioblastoma. [file Image_8.tif]

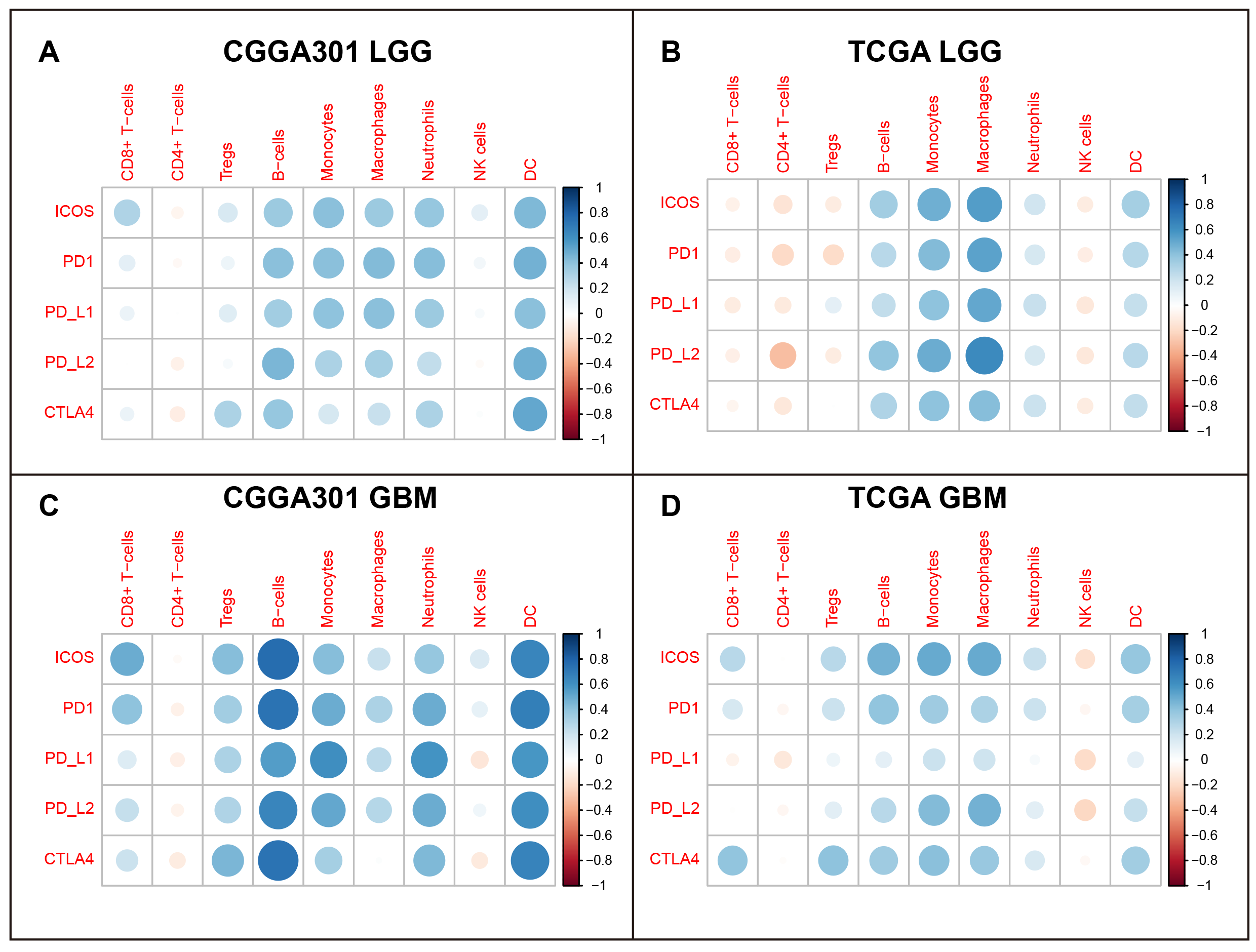

Supplement: Supplementary Figure 9 — Comparison of cell fraction correlation between ICOS and other checkpoints in CGGA301 and TCGA datasets. [file Image_9.tif]

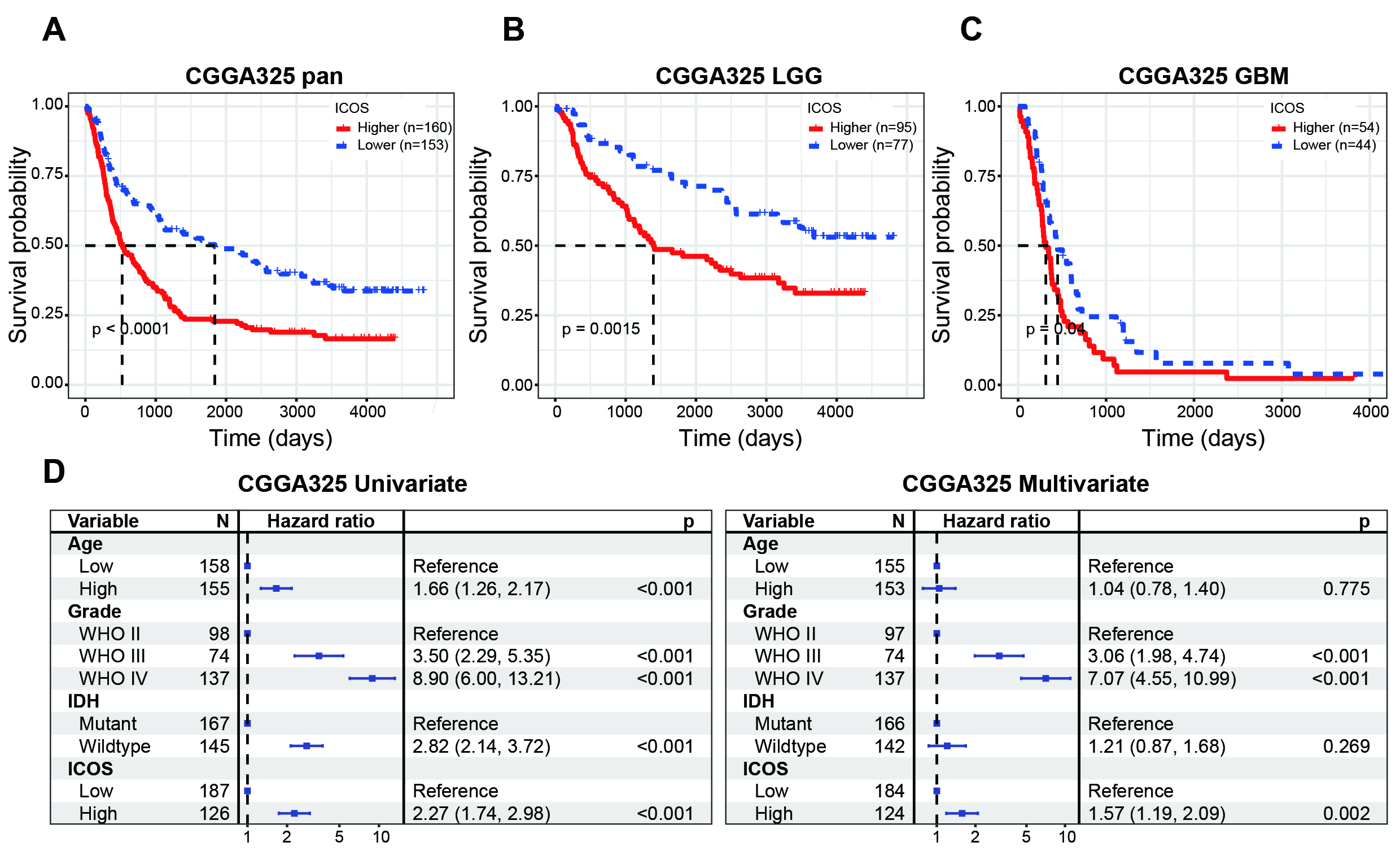

Supplement: Supplementary Figure 10 — Survival analysis and Cox proportional hazards regression analysis according to ICOS expression in CGGA325 dataset. (A–C) Survival analysis in CGGA325 dataset. (D) Univariate and multivariate Cox regression model in CGGA325 dataset. [file Image_10.tif]
